# Chemical Vapor Deposition of High‐Quality Large‐Sized MoS_2_ Crystals on Silicon Dioxide Substrates

**DOI:** 10.1002/advs.201600033

**Published:** 2016-03-31

**Authors:** Jianyi Chen, Wei Tang, Bingbing Tian, Bo Liu, Xiaoxu Zhao, Yanpeng Liu, Tianhua Ren, Wei Liu, Dechao Geng, Hu Young Jeong, Hyeon Suk Shin, Wu Zhou, Kian Ping Loh

**Affiliations:** ^1^ Centre for Advanced 2D MaterialsNational University of Singapore6 Science Drive 2Singapore117546Singapore; ^2^ Department of ChemistryNational University of Singapore3 Science Drive 3Singapore117546Singapore; ^3^ Department of ChemistryUlsan National Institute of Science and Technology (UNIST)UNIST‐gil 50Ulsan689‐798Republic of Korea; ^4^ Department of Energy EngineeringUlsan National Institute of Science and Technology (UNIST)UNIST‐gil 50Ulsan689‐798Republic of Korea; ^5^ UNIST Central Research Facilities (UCRF)Institute of Basic ScienceUlsan National Institute of Science and Technology (UNIST)UNIST‐gil 50Ulsan689‐798Republic of Korea; ^6^ Materials Science and Technology DivisionOak Ridge National LaboratoryOak RidgeTN37831USA

**Keywords:** chemical vapor deposition, high quality, large size, molybdenum disulfide, silicon dioxide

## Abstract

**Large‐sized MoS_2_ crystals** can be grown on SiO_2_/Si substrates via a two‐stage chemical vapor deposition method. The maximum size of MoS_2_ crystals can be up to about 305 μm. The growth method can be used to grow other transition metal dichalcogenide crystals and lateral heterojunctions. The electron mobility of the MoS_2_ crystals can reach ≈30 cm^2^ V^−1^ s^−1^, which is comparable to those of exfoliated flakes.

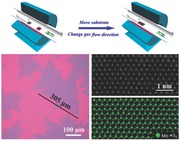

Post‐graphene, there is intense interest in transition metal dichalcogenide (TMD) owing to its unique properties of large spin‐orbit coupling and a bandgap, which offer new possibilities in electronics and valleytronics.^1^, ^2^, ^3^ MoS_2_ is one of the most widely studied TMDs. It is a layered 2D material in which the transition metal Mo atoms are sandwiched between two planes of S atoms.^4^, ^5^ Bulk MoS_2_ crystals have an indirect bandgap of ≈1.29 eV, however its monolayer exhibits a direct bandgap of ≈1.8 eV.^6^ Monolayer MoS_2_ gives rise to strong photo‐ and electro‐luminescence due to the direct bandgap.^7^, ^8^ According to previous reports,^9^, ^10^ the room temperature mobility of MoS_2_ can reach ≈410 cm^2^ V^−1^ s^−1^ with a high on/off ratio of 10^8^. The excellent optical and electrical properties render MoS_2_ an attractive candidate for applications as transistor, photodetectors, photovoltaic cells, piezoelectricity, and spintronic devices.^9^, ^10^, ^11^, ^12^, ^13^, ^14^

To date, many efforts have been developed to prepare monolayer MoS_2_, including micromechanical exfoliation, chemical exfoliation, hydrothermal synthesis, and physical vapor deposition.^2^, ^15^, ^16^ Among these methods, chemical vapor deposition (CVD) is most promising in terms of scalability, simple operation and low cost, and has been used to grow various 2D materials directly on dielectric substrates.^17^, ^18^, ^19^, ^20^, ^21^, ^22^, ^23^, ^24^, ^25^, ^26^ However, similar to other TMDs,^19^, ^20^, ^21^ CVD‐grown continuous MoS_2_ film suffer from a high density of rotational domain boundaries.^27^, ^28^ There is increasing evidence that defects inherent in polycrystalline films prevent the full potential of 2D materials to be realized.^29^, ^30^ Therefore it is very important to grow large MoS_2_ single crystals to minimize the presence of defects arising from boundaries.

Recently Chen. et al. studied the role of oxygen on the growth of MoS_2_, and obtained large‐sized crystals by a low‐pressure CVD method.^31^ However the introduction of oxygen into reaction system is dangerous, and is not an essential prerequisite to the growth of large‐sized crystals. Here, we found that, by controlling the growth process under ambient pressure using a two‐stage CVD method, the nucleation density of MoS_2_ can be significantly reduced, thus also forming large‐sized crystals. Unlike expensive sapphire, the direct growth of MoS_2_ crystals on low‐cost SiO_2_/Si substrates is more compatible with current Si processing techniques for fabrication of electronic devices. The as‐made MoS_2_ grains are monolayer crystals. Their maximum size can reach up to ≈305 μm, comparable to that of previous reports.^27^, ^28^, ^29^, ^30^ Raman spectroscopy, transmission electron microscopy (TEM) and field effect transistor (FET) measurements indicate that these crystals have excellent crystallinity and electronic properties. The electron mobility can reach about 30 cm^2^ V^−1^ s^−1^ with an on/off ratio above 10^6^. The growth method can also be used to grow other TMD crystals such as MoSe_2_ and WS_2_. We further use the method for epitaxial growth of lateral MoS_2_/WS_2_ heterojunctions. The atomically sharp in‐plane junctions have excellent current rectification behavior, which is important for potential applications in electronics and optoelectronics.

The CVD process was performed under ambient pressure and the detailed growth procedures are described in **Figure** **1**a and Figure S1 in the Supporting Information. According to previous reports,^32^, ^33^ to realize the growth of large‐sized 2D materials, it is important to decrease the nucleation density and increase the growth rate of the nuclei. To achieve that, our strategy is to compartmentalize the growth process: separating the induction stage from the growth stage. The induction stage is needed to isolate the growth substrate before the targeted high temperature and equilibrium evaporation rate is reached, since the nucleation and growth can occur during heating stage (Figure S2, Supporting Information), resulting in the formation of a high density of smaller crystals. Briefly, MoS_2_ crystals were grown on SiO_2_/Si substrates with sulfur (S) and molybdenum trioxide (MoO_3_) as the precursors using a modified CVD system (Figure 1a). MoO_3_ powder (about 1.0 mg) was placed on a quartz slide, which were located in the heating zone center of the furnace. A smaller quartz test tube, containing 0.8 g of S, was located upstream, and the open end exposed to the center of the furnace. Unlike the widely used method in which substrates are put face‐down above the MoO_3_ source, our SiO_2_/Si substrate was put at the downstream side (the left picture in Figure 1a). During the induction phase, the furnace temperature was raised to 850 °C and 200 sccm Ar was introduced in a direction flowing away from the substrate to prevent any unintentional nucleation and growth of MoS_2_ crystals. When the targeted growth temperature and equilibrium vapor pressure in the growth zone was reached, the SiO_2_/Si substrate was rapidly introduced into the growth zone where MoO_3_ sources were located by using a homemade setup. Meanwhile, the direction of gas flow was reversed and flow rate set to 20 sccm to allow reactants to flow to the substrate (the right picture in Figure 1a). The growth time was about 10 min (Figure S3, Supporting Information). Compared with the general one‐stage growth process, the physical segregation of the CVD process into induction and growth stages allows the substrate to be exposed to the targeted high temperature and vapor pressure quickly, thus avoiding undesired nucleation during the ramp up period (Figure S4, Supporting Information).

**Figure 1:**
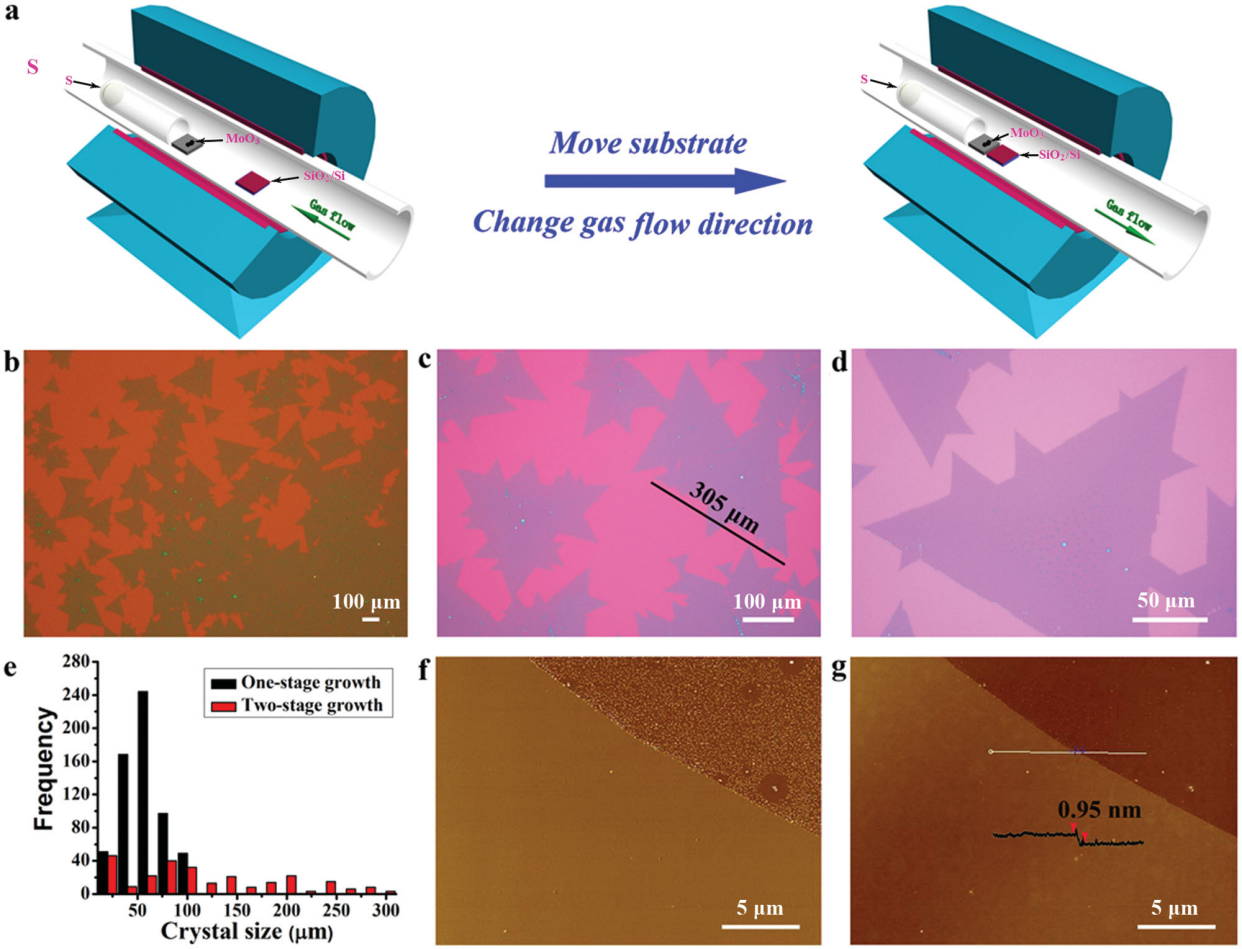
a) Schematics for the CVD synthesis of large‐sized MoS_2_ crystals. b−d) Typical optical images of triangular MoS_2_ crystals. e) Size distribution of MoS_2_ crystals obtained by different methods. f) AFM phase and g) height images of MoS_2_ monolayer.

Figure 1b show the optical image of the as‐grown MoS_2_ crystals. For comparison, we also show the optical image of MoS_2_ crystals grown by a one‐stage method (Figure S5, Supporting Information). Due to the optical contrast, it is straightforward to identify MoS_2_ domains from the SiO_2_ substrate. Similar to previous reports,^27^, ^28^, ^29^, ^30^ adjacent MoS_2_ crystals have coalesced to form a film. The crystal size of MoS_2_ crystals ranges from several tens to hundreds of micrometers. Discrete smaller crystals show a regular triangular morphology, while larger crystals easily form twin crystals with smaller crystals (Figure 1c). An enlarged image of MoS_2_ crystals, shown in Figure 1d, displays a uniform color contrast on the SiO_2_/Si substrate, indicating that the crystals are of uniform thickness. Figure 1e shows a size histogram of MoS_2_ crystals observed using optical microscopy. The majority of the MoS_2_ crystals are one order of magnitude in area than those produced using one‐stage method.

To identify the number of layers for our MoS_2_ sample, the edges of crystals are measured using atomic force microscopy (AFM). Figure 1f,g are typical tapping mode AFM images of a MoS_2_ crystal. The sharper, straighter edge may indicate the formation of molybdenum zigzag (Mo‐zz) edge structure.^29^ The homogeneity of film thickness is evidenced by color homogeneity. Height profiles across MoS_2_ edge samples (Figure 1g) show that thickness of our sample is about 0.95 nm, corresponding to monolayer MoS_2_.

MoS_2_ crystals were further characterized by using TEM, selected area electron diffraction (SAED), scanning transmission electron microscope (STEM). These techniques provide important information about the structure and quality of MoS_2_ crystals as detailed below. After the MoS_2_ crystals were transferred to a copper grid, the layer count on the edge of the image (**Figure** **2**a) indicated that the crystal is monolayer MoS_2_. The high‐magnification TEM image in Figure 2b shows a honeycomb arrangement of atoms, and the selected SAED pattern in Figure 2c displays one set of hexagonal symmetrical patterns, indicating the hexagonal lattice structure of MoS_2_ crystals.^34^ The atomic structure of MoS_2_ crystals was studied by annular dark field (ADF) imaging (Figure 2d). The corresponding atomic model is shown in Figure 2e. Because the signal intensity in the STEM–ADF image is directly related to the average atomic number (Z), STEM–ADF image can thus be used to visualize the spatial distribution of Mo ans S due to their different image contrast levels.^35^ The sharp atomic images indicate that our samples have a high crystalline quality, in accordance with previous reports.

**Figure 2:**
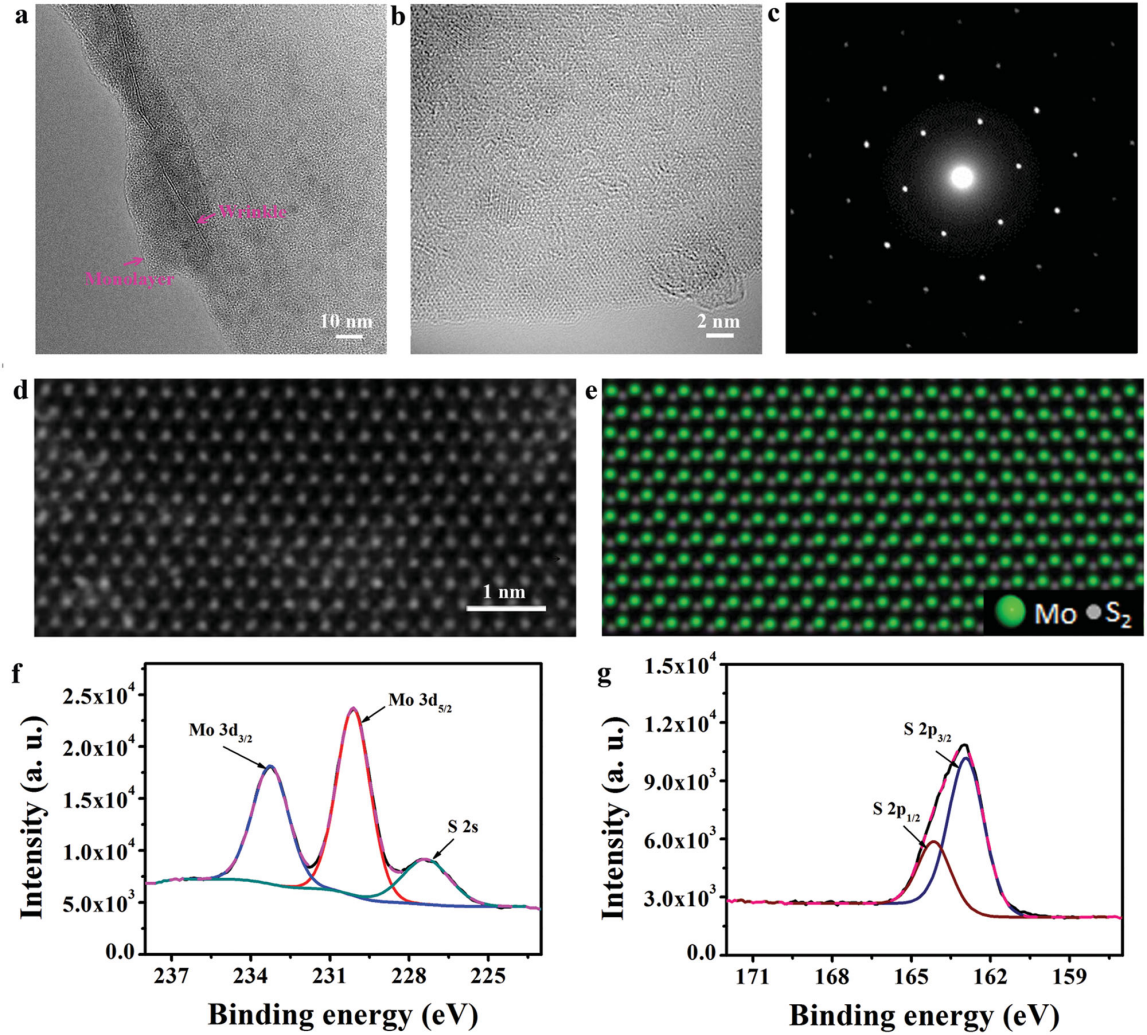
a,b) High‐resolution TEM images of MoS_2_ crystals. c) SAED pattern of MoS_2_ crystals. d) High‐magnification STEM ADF image of MoS_2_ crystals. e) The atomic models correspond to the structure in (e). f,g) XPS spectra of MoS_2_ crystals showing f) Mo 3d and g) S 2p peaks.

X‐ray photoelectron spectroscopy (XPS) was used to examine the elemental composition and bonding of MoS_2_ samples. Only four elements (Mo, S, O, and Si) are observed in the spectra (Figure S6, Supporting Information), confirming that MoS_2_ was directly synthesized on SiO_2_/Si substrates. The Mo 3*d* and S 2*p* peaks provide important information about the stoichiometry and bonding of the MoS_2_ crystals (Figure 2f,g). The Mo 3*d*_3/2_ and 3*d*_5/2_ peaks are located at ≈230.0 and ≈233.2 eV, respectively, while the S 2*p*_1/2_ and S 2*p*_3/2_ peaks are located at ≈164.0 and ≈162.9 eV, respectively. These peak positions are consistent with the reported values for 2H‐MoS_2_ crystals.^34^ The positions of the Mo peaks indicate the reduction of Mo from Mo^6+^ (MoO_3_) to Mo^4+^ (MoS_2_). The Mo/S ratio obtained from Mo 3*d* and S 2*p* XPS is about 1:1.97, suggesting that the CVD MoS_2_ film is stoichiometric with some S vacancies,^36^ which were reported as the dominant point defect in CVD‐grown MoS_2_.^37^

Raman and photoluminescence (PL) microscopy are powerful methods for the characterization of crystal quality and bandgap in TMD materials. Typical monolayer MoS_2_ crystals were characterized with Raman and PL using a laser wavelength of 532 nm. **Figure** **3**a shows the Raman spectrum of the MoS_2_ sample. The monolayer sheet exhibits two characteristic Raman bands at 400.2 and 383.4 cm^−1^, corresponding to the A_1g_ and E^1^_2g_ modes of monolayer MoS_2_ crystals,^28^, ^34^ and their full‐width‐half‐maximum (FWHM) values are about 6.8 and 3.8 cm^−1^, respectively. The PL spectrum (Figure 3b) shows highly distinct photoluminescence peaks at ≈623 and 673 nm, corresponding to the A1 and B1 direct excitonic transitions of MoS_2_ monolayer, respectively.^8^, ^28^ To probe the micro‐scale structure of the crystal, we also conducted Raman and PL mapping centered at ≈400.1 cm^−1^ (the A_1g_ mode), ≈383.4 cm^−1^ (the E^1^_2g_ mode) and ≈673 nm (the PL mode), as shown in Figure 3c–e. The uniform color intensity observed suggests that the MoS_2_ crystal is uniform in thickness.

**Figure 3:**
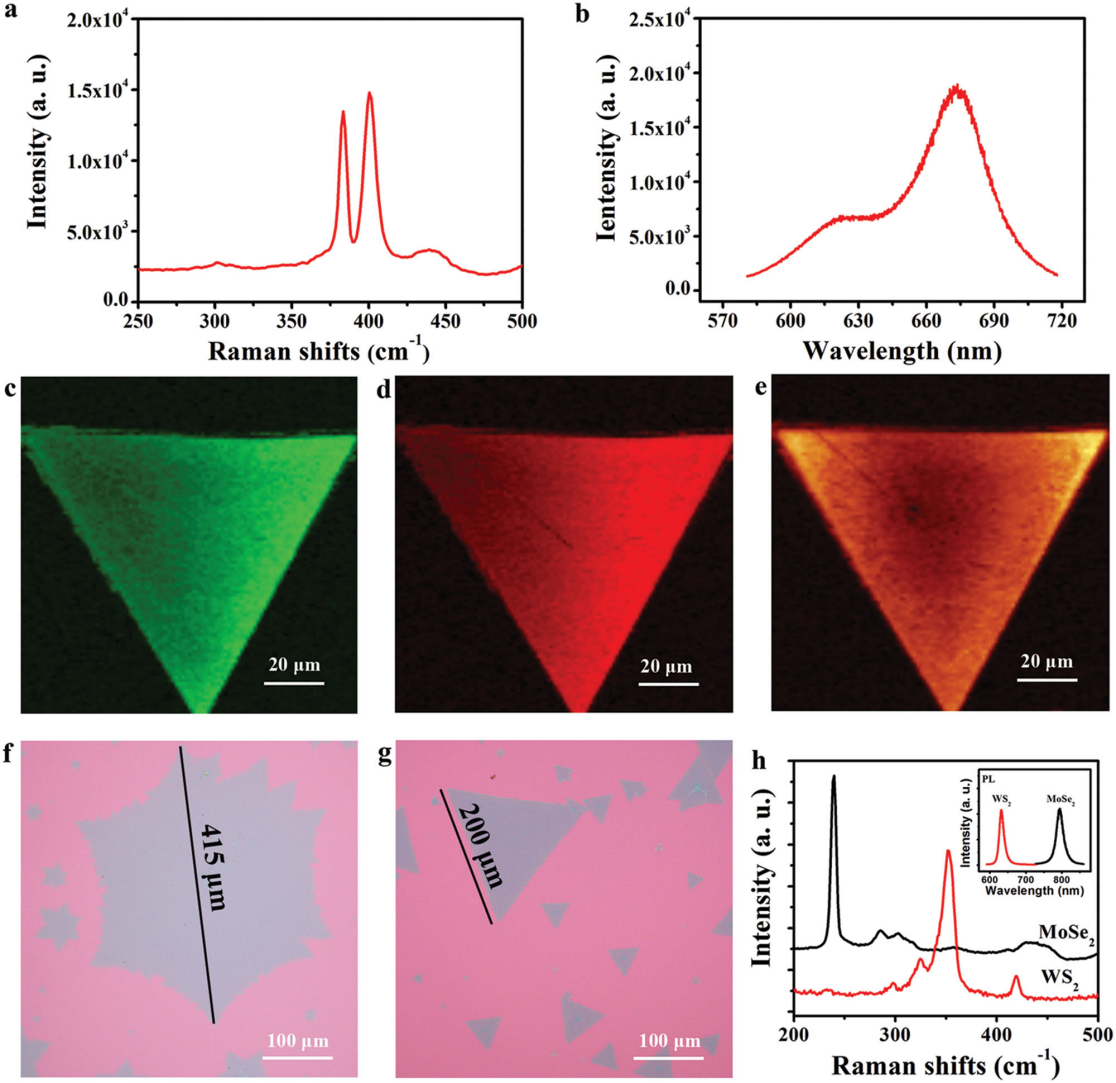
a) Raman and b) PL spectra of MoS_2_ monolayer. c−e) Raman and PL mapping centered at f) ≈400.1 cm^−1^, g) ≈383.4 cm^−1^, and h) ≈673 nm. Optical images of f) large‐sized hexagonal MoSe_2_ and g) triangular WS_2_ crystals. h) Raman spectra of MoSe_2_ and WS_2_ monolayer. The inset shows PL spectra of MoSe_2_ and WS_2_ monolayer.

To assess the generality of the method for growing other TMDs crystals, we also tried to synthesize MoSe_2_ and WS_2_ crystals using a similar strategy. MoSe_2_ and WS_2_ were grown using MoO_3_, Se and WO_3_, S powders as the source precursors respectively. The difference is that a small quantity of H_2_ (1.5 sccm) is required to enhance the selenization reaction of MoO_3_ during the growth of MoSe_2_ crystals. The introduction of H_2_ also changes the relative edge free energy of Se edges and Mo edges, thus forming hexagonal crystals under suitable conditions.^38^, ^39^ Nevertheless, we have obtained large‐sized MoSe_2_ and WS_2_ crystals on SiO_2_/Si substrates. (Figure 3f,g). Figure 3h shows the Raman sprectra of these MoSe_2_ and WS_2_ crystals. The A_1g_ and E_2g_ modes of MoSe_2_ single‐layer are located at ≈239.7 cm^−1^ (A_1g_), 286.2 cm^−1^ (E_2g_) respectively, while the A_1g_ and E_2g_ modes of WS_2_ single‐layer are located at 418.8 and 352. 3 cm^−1^ respectively.^40^, ^41^ The PL spectra (inset) shows the characteristic emission peaks corresponding to the emission of MoSe_2_ (≈794 nm) and WS_2_ (≈632 nm) monolayer.^42^, ^43^ These results indicate that these crystals are monolayer crystals with perfect optical properties.

To investigate the electronic quality of the CVD‐grown MoS_2_ crystals, we measured the electrical transport properties. **Figure** **4**a shows a schematic diagram of MoS_2_ FETs fabricated on SiO_2_/Si substrates using Ti/Au as the source–drain (S–D) electrodes and a doped silicon substrate as the back gate. The typical *I*−*V* characteristics for a MoS_2_ FET measured in nitrogen atmosphere is shown in Figure 4b. A linear *I*_DS_−*V*_DS_ relationship is clearly observed, indicating that ohmic contacts were formed at the source and drain electrodes. The transfer characteristics (drain current *I*_DS_ vs gate voltage *V*_G_) of the MoS_2_ device are shown in Figure 4c. The *I*_DS_ value increases monotonically with increasing *V*_G_, which is indicative of n‐type semiconducting behavior. The field‐effect mobility of this MoS_2_ FET was estimated to be ≈28 cm^2^ V^−1^ s^−1^ with an on/off rario above 10^6^. The mobilities of all the 20 devices we measured are in the range of 1−30 cm^2^ V^−1^ s^−1^, comparable to prevous reports.^28^, ^29^, ^30^ The mobility could be improved by high‐k top gate dielectrics and interface engineering.^10^, ^31^, ^44^

**Figure 4:**
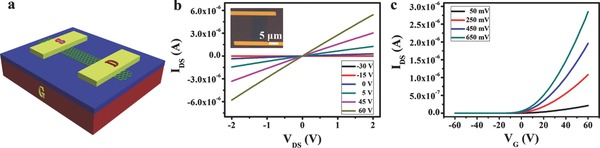
a) Schematic diagram of MoS_2_‐based device. MoS_2_ ribbon is obtained by EBL and vapor‐phase etching techniques. b) Current (*I*_SD_)/voltage (*V*_SD_) output characteristics of a MoS_2_ FET device at various back gate voltages. The inset shows the optical image of the device. c) Transfer curves (*I*_DS_–V_G_) of a back‐gated MoS_2_ device at various source–drain voltages.

Beyond the growth of single crystals, we have also realized the growth of WS_2_ crystals along the edges of MoS_2_ crystals, and formed MoS_2_/WS_2_ lateral heterojunctions by our method (**Figure** **5**a and Figure S7, Supporting Information). Observation under STEM indicates that the lateral interface is atomically sharp (Figure 5b), without extensive (WMo)S_2_ alloying region.^45^, ^46^, ^47^, ^48^ The chemical modulation cross the lateral heterostructure is confirmed by elemental mapping using electron energy‐loss spectroscopy (EELS) imaging (Figure 5c−e). The Raman and PL mapping of the characteristic peaks and peaks of WS_2_ and MoS_2_ also revealed the structural modulation between MoS_2_ and WS_2_ (Figure 5f,g and Figure S8, Supporting Information). The lateral stitching of MoS_2_ monolayer and WS_2_ monolayer has formed an in‐plane heterojunction. The electrical transport across the interface of monolayer MoS_2_/WS_2_ in‐plane heterojunctions was measured (Figure S9, Supporting Information). The forward bias current is higher than the reverse current, suggesting reasonably good rectification across this in‐plane heterojunction.

**Figure 5:**
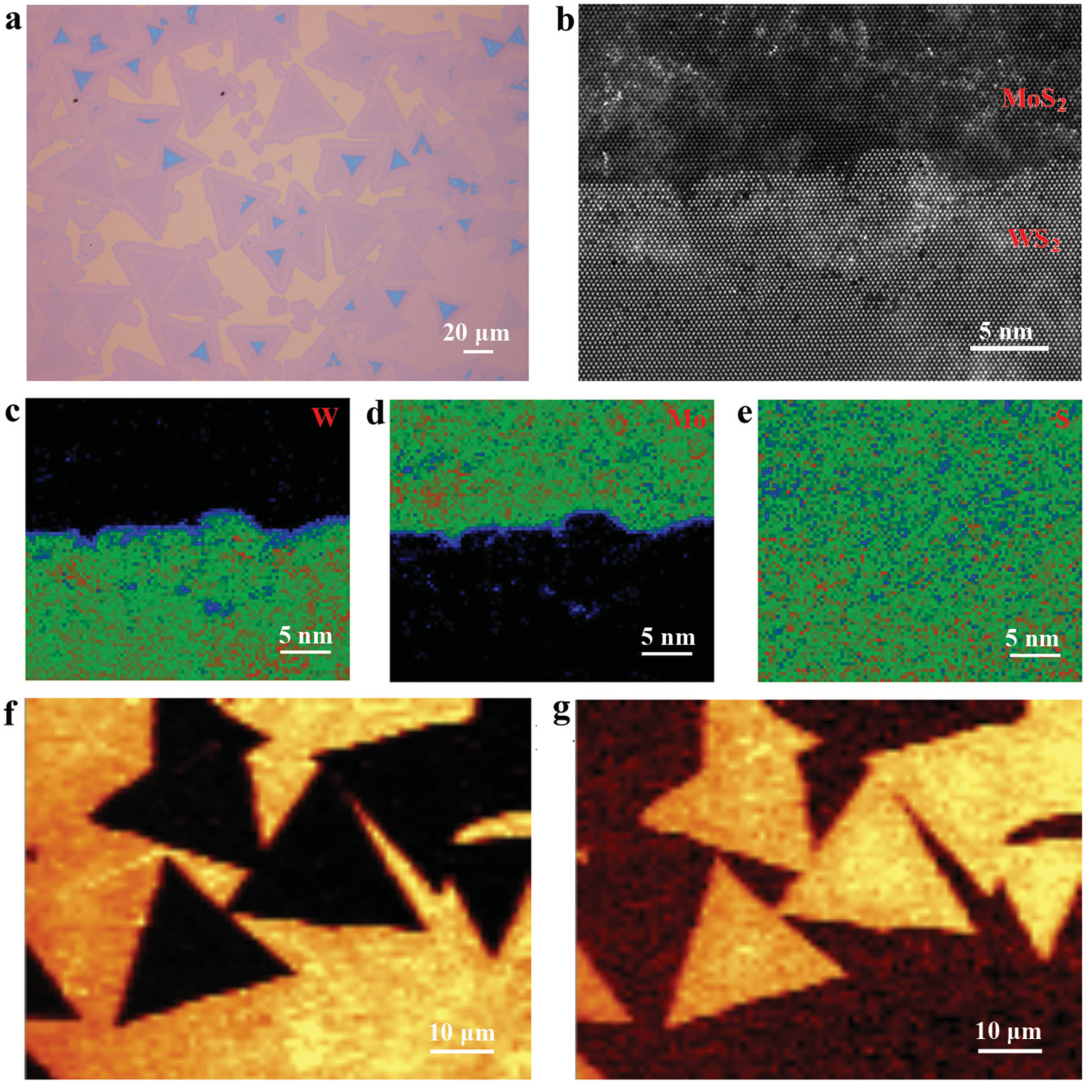
a) Optical image of MoS_2_/WS_2_ in‐plane heterojunctions. b) High‐magnification STEM ADF image of the lateral MoS_2_/WS_2_ heterojunction. Elemental mapping images of c) Mo, d) W, and e) S atoms. Raman mapping of the characteristic E^1^_2g_ peaks of WS_2_ centered at f) ≈352.3 cm^−1^ and g) MoS_2_ centered at ≈383.4 cm^−1^.

In summary, we have successfully realized the growth of large‐sized, high‐quality MoS_2_ crystals. The nucleation density of crystals can be decreased by separating the induction stage from the growth stage, and the maximum size of MoS_2_ crystals can reach about 305 μm. Electrical transport measurements indicate that the MoS_2_ crystals have electron mobility up to about 30 cm^2^ V^−1^ s^−1^, comparable to those of exfoliated flakes and CVD synthetic crystals. The growth method can also be used to grow other TMD crystals such as MoSe_2_ and WS_2_, suggesting the universality of of the method. In addition, we have also demonstrated the lateral epitaxy growth of MoS_2_/WS_2_ in‐plane heterojunctions. These junctions have atomically sharp interface with a good rectification characteristic.

## Experimental Section

*Preparation of MoS_2_ Crystals*: MoS_2_ crystals were grown on dielectric substrates by using a modified ambient pressure CVD method. A little MoO_3_ powder (about 1.0 mg) was placed on growth substrate which was introduced into the heating zone center of the 2 in. furnace. A smaller quartz tube with one end sealed containing 0.8 g of sulfur powder was located upstream, and the open end extended to the center of the furnace. The SiO_2_/Si growth substrate was put at the downstream side. The furnace temperature was raised to 850 °C and 200 sccm Ar was introduced in a direction flowing away from the substrate. The SiO_2_/Si substrate was moved and made close to MoO_3_ sources. Meanwhile, the direction of flowing gas was chaged and the gas flow at 20 sccm was controlled . After stabilizing the system for 10 min, the furnace was cooled to room temperature.

*Characterization*: Optical images were obtained using a Nikon ECLIPSE LV100D microscopy. AFM images were performed using a Bruker Dimension FastScan Atomic Force Microscope in the tapping mode. Raman spectra were recorded at room temperature using a WITec Raman Microscope with laser excitation at 532 nm. TEM was performed with FEI Titan transmission electron microscope operated at 80 kV. STEM imaging and EELS analysis were performed on an aberration‐corrected Nion UltraSTEM‐100 operating at 60 kV. XPS analysis was carried out on an Omicron EAC2000‐125 analyzer. Base pressure during analysis was 10^−9^ Torr. An Al Kα monochromatized radiation (hν = 1486.6 eV) was employed as the X‐ray source.

Device *and Electrical Measurements*: Triangular MoS_2_ crystals were etched into ribbons by electron beam lithography (EBL) and oxygen plasma. FETs were fabricated on SiO_2_/Si wafers with Ti/Au (5/50 nm) as source–drain electrodes and the doped silicon substrate as the back gate. The FET characteristics were measured in N_2_ at room temperature. A Keithley 4200SC semiconductor parameter analyzer was used to measure the electrical characteristics of the devices.

## Supporting information

As a service to our authors and readers, this journal provides supporting information supplied by the authors. Such materials are peer reviewed and may be re‐organized for online delivery, but are not copy‐edited or typeset. Technical support issues arising from supporting information (other than missing files) should be addressed to the authors.

Supplementary
