# Supplementary material for: Chemical Vapor Deposition of High‐Quality Large‐Sized MoS2 Crystals on Silicon Dioxide Substrates
Source: Adv Sci (Weinh). 2016 Mar 31;3(8):1500033. doi: 10.1002/advs.201600033 (PMC5071677; doi:10.1002/advs.201600033)
Supplement: Supplementary file 1 — Supplementary [file ADVS-3-0l-s001.pdf]

Copyright WILEY-VCH Verlag GmbH & Co. KGaA, 69469 Weinheim, Germany, 2016.

Supporting Information

for *Adv. Sci.,* DOI: 10.1002/advs.201600033

Chemical Vapor Deposition of High-Quality Large-Sized MoS_2 Crystals on Silicon Dioxide Substrates

*Jianyi Chen, Wei Tang, Bingbing Tian, Bo Liu, Xiaoxu Zhao, Yanpeng Liu, Tianhua Ren, Wei Liu, Dechao Geng, Hu Young Jeong, Hyeon Suk Shin, Wu Zhou, and Kian Ping Loh**

Copyright WILEY-VCH Verlag GmbH & Co. KGaA, 69469 Weinheim, Germany, 2013.

Supporting Information

Chemical Vapor Deposition of High-Quality Large-Sized MoS_2 Crystals on Silicon Dioxide Substrates

*Jianyi Chen, Wei Tang, Bingbing Tian, Bo Liu, Xiaoxu Zhao, Yanpeng Liu, Tianhua Ren, Wei Liu, Dechao Geng, Hu Young Jeong, Hyeon Suk Shin, Wu Zhou, Kian Ping Loh **

1: Growth procedures of large-sized MoS_2 crystals

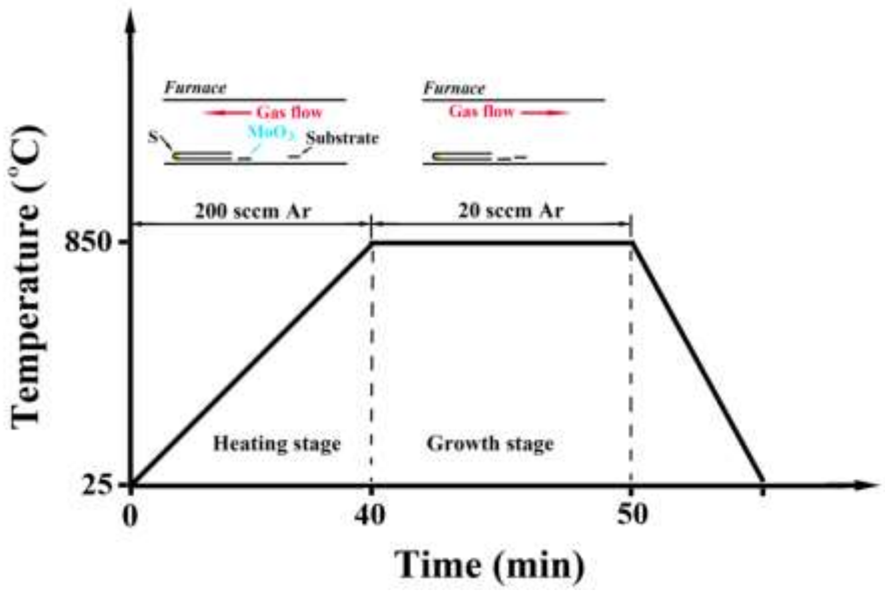

Figure S1 Growth procedures of large-sized MoS_2 crystals.

2: Optical images of MoS_2 crystals

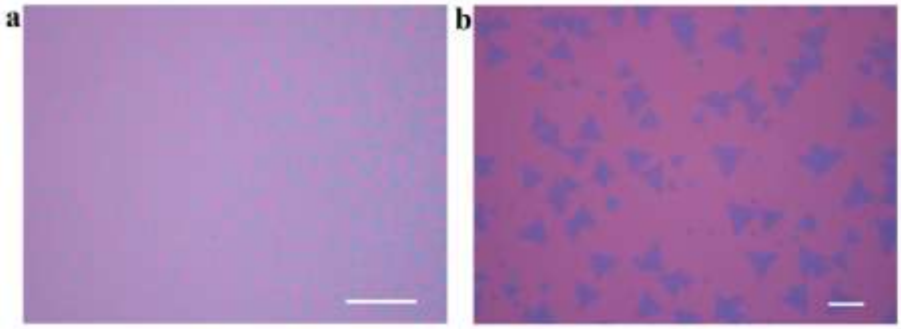

Figure S2 Optical images of MoS_2 crystals. The growth is performed at 600 °C for 10 min using the general method. The substrate is close to MoO_3 source without moving during the

growth process. a) Scale bar 100 μm. b) Scale bar 10 μm. The experiment indicated that growth of MoS_2 crystals can occurred as low as 600 ˚C.

3: Optical images of MoS_2 crystals with different growth time

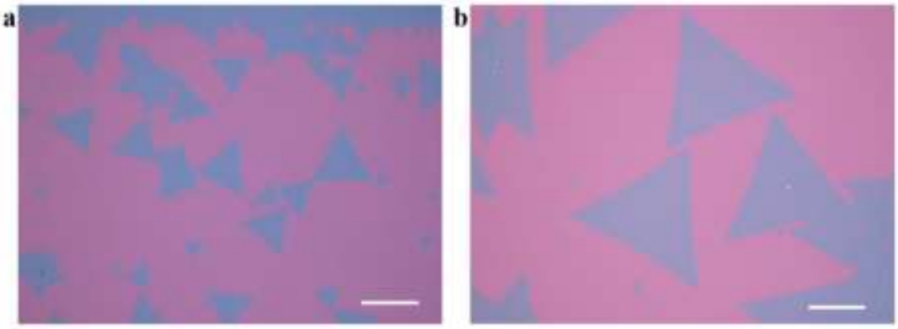

Figure S3 Optical images of MoS_2 crystals with different growth time. a) 5 min. b) 20 min. Scale bar 100 μm.The size of monolayer MoS_2 crystals could not be increased by extending growth time, while long growth time easily caused the depostion of multilayer crystals on the surface.

4: Optical images of SiO_2/Si substrate

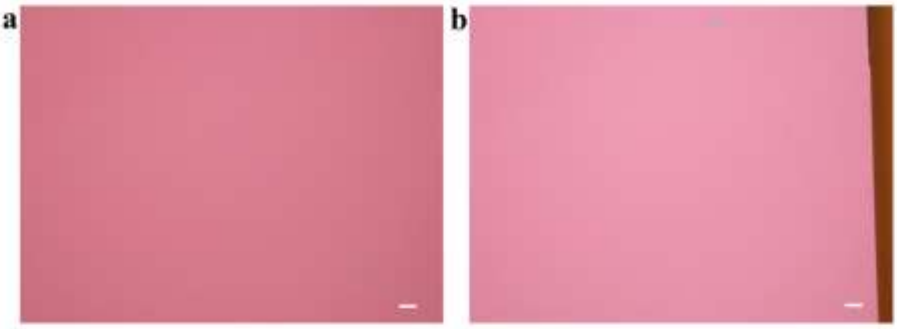

Figure S4 Optical images of SiO_2/Si substrates. The growth is performed using the modified CVD method at 850 ˚C for 5 min. The substrate is not removed. We only saw few MoS_2 crystals at the edge of the SiO_2/Si substrate (b). Scale bar 100 μm.

5: Optical image of MoS_2 crystals by one-stage method

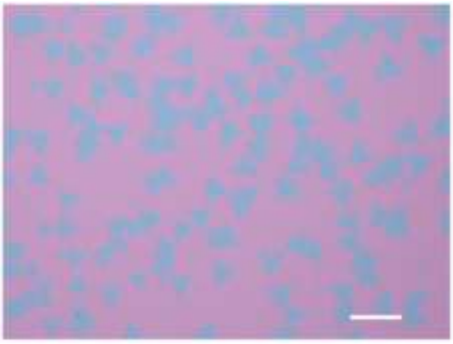

Figure 5 Optical image of MoS_2 crystals by one-stage method. Scale bar 100 μm.

6: Full XPS spectrum of MoS_2 grown on a SiO_2/Si substrate

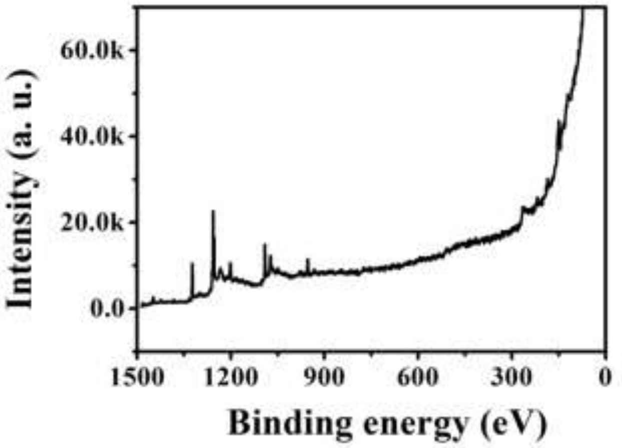

Figure S6 Full XPS spectrum of MoS_2 grown on a SiO_2/Si substrate.

7: Optical image of MoS_2/WS_2 heterostructure with larger MoS_2 regions

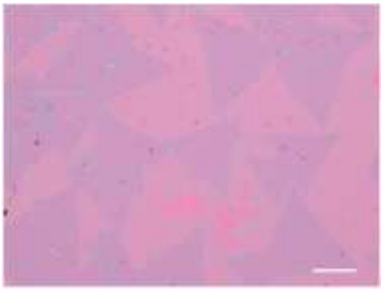

Figure S7 Optical image of MoS_2/WS_2 heterojunctions with larger MoS_2 regions. Scale bar 100 μm. MoS_2/WS_2 heterojunctions were grown on dielectric substrates by a two-stage method similar to the growth of MoS_2 crystals (See the method) with the difference that the

clean SiO_2/Si substrates were replaced by SiO_2/Si substrates on which MoS_2 crystals were pre-grown. The amount of WO_3 powder was increased to 10 mg to enhance the supply of W source, and the temperature was controlled at 700 ˚C to realize the growth of in-plane heterojunctions.

8: Raman and PL characterizations of in-plane heterojunctions.

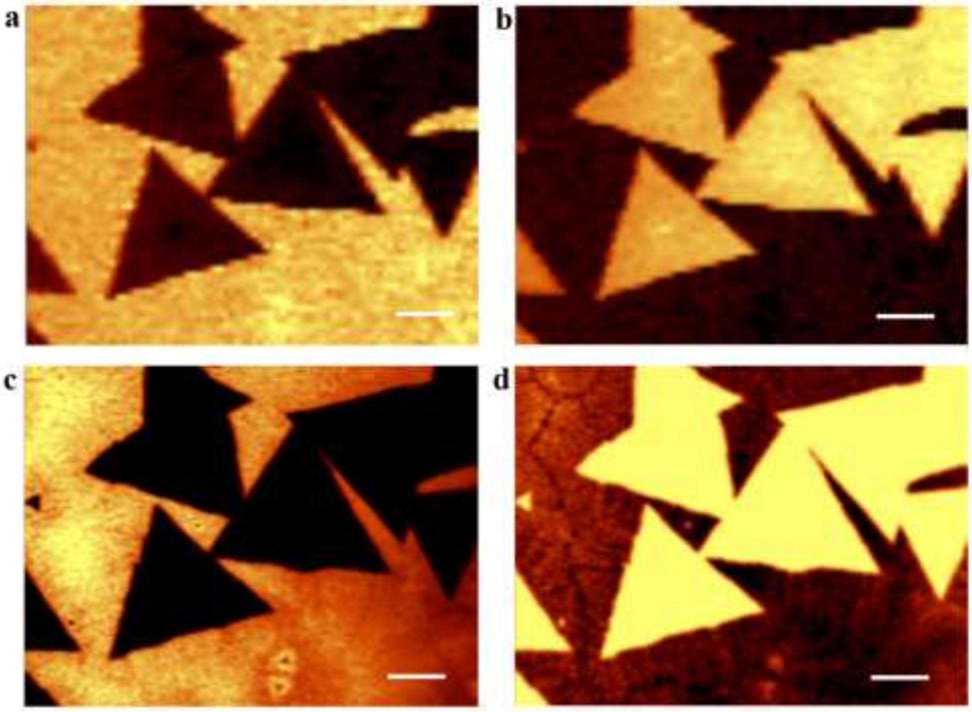

Figure S8 Raman and PL characterizations of in-plane heterojunctions. a) Raman mapping of the A_{1g} peak of WS_2 (centered at ~418.8 cm^{-1}). d) Raman mapping of the A_{1g} peak of MoS_2 (centered at 400.2 cm^{-1}). c) PL mapping of the characteristic peak of WS_2 (centered at ~632 nm). d) PL mapping of the characteristic peaks of MS_2 (centered at ~673 nm). Scale bar 10 μm.

9: Electrical property of lateral MoS_2/WS_2 heterojunction diode

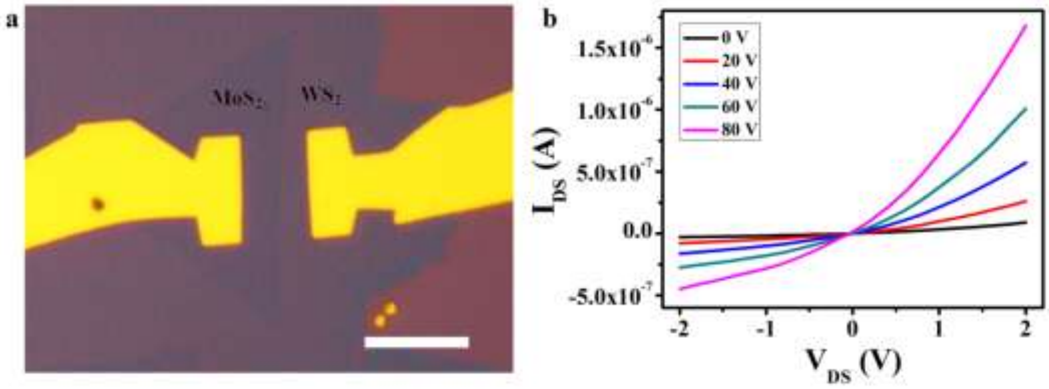

Figure S9 Electrical property of lateral MoS_2/WS_2 heterojunction diode. a) Optical image of a lateral MoS_2/WS_2 heterojunction diode. Scale bar b) Gate-tunable characteristics of a lateral MoS_2/WS_2 heterojunction diode. Scale bar 10 μm.
